# Antileishmanial activity of the essential oil from *Tetradenia riparia* obtained in different seasons

**DOI:** 10.1590/0074-02760150290

**Published:** 2015-12

**Authors:** Bruna Muller Cardoso, Tatiane França Perles de Mello, Sara Negrão Lopes, Izabel Galhardo Demarchi, Daniele Stefani Lopes Lera, Raíssa Bocchi Pedroso, Diogenes Aparício Cortez, Zilda Cristiani Gazim, Sandra Mara Alessi Aristides, Thais Gomes Verzignassi Silveira, Maria Valdrinez Campana Lonardoni

**Affiliations:** 1 Universidade Estadual de Maringá, Departamento de Análises Clínicas e Biomedicina, Programa de Pós-Graduação em Ciências da Saúde, Maringá, PR, Brasil; 2 Universidade Estadual de Maringá, Departamento de Farmácia e Farmacologia, Programa de Pós-Graduação em Ciências Farmacêuticas, Maringá, PR, Brasil; 3 Universidade Paranaense, Umuarama, PR, Brasil

**Keywords:** Tetradenia riparia, essential oil, Leishmania (Leishmania) amazonensis, antileishmanial activity, nitric oxide

## Abstract

The herbaceous shrub *Tetradenia riparia* has been traditionally used to treat inflammatory and infectious diseases. Recently, a study showed that *T. riparia* essential oil (TrEO) obtained in summer has antileishmanial effects, although these results could be influenced by seasonal variation. This study evaluated the activity of the TrEO obtained in different seasons against *Leishmania (Leishmania) amazonensis*, in vitro and in vivo. The compounds in the TrEO were analysed by gas chromatography-mass spectrometry; terpenoids were present and oxygenated sesquiterpenes were the majority compounds (55.28%). The cytotoxicity and nitric oxide (NO) production were also tested after TrEO treatment. The TrEO from all seasons showed a 50% growth inhibitory concentration for promastigotes of about 15 ng/mL; at 30 ng/mL and 3 ng/mL, the TrEO reduced intracellular amastigote infection, independently of season. The TrEO from plants harvested in summer had the highest 50% cytotoxic concentration, 1,476 ng/mL for J774.A1 macrophages, and in spring (90.94 ng/mL) for murine macrophages. NO production did not change in samples of the TrEO from different seasons. The antileishmanial effect in vivo consisted of a reduction of the parasite load in the spleen. These results suggest that the TrEO has potential effects on *L. (L.) amazonensis*, consonant with its traditional use to treat parasitic diseases.

Leishmaniases are chronic diseases caused by parasites of the genus*Leishmania* and affect more than 12 million people in 88 countries. The worldwide incidence is about 1.5-2.0 million, and 350 million people live in areas where they are at risk of infection (WHO 2010). Clinical manifestations of this group of diseases include localised cutaneous leishmaniasis, diffuse cutaneous leishmaniasis, mucocutaneous leishmaniasis and visceral leishmaniasis, which may be fatal if untreated (Reithinger et al. 2007, Santos et al. 2008b). In Brazil, cutaneous leishmaniasis caused mainly by *Leishmania (Leishmania) amazonensis* and*Leishmania (Viannia) braziliensis* occurs throughout the country. Leishmaniasis is considered a public-health problem and one of the neglected tropical diseases (Santos et al. 2008b).

The first-choice drugs for the treatment of leishmaniasis are the pentavalent antimonials such as N-methylglucamine antimoniate (Glucantime^®^, Sanofi-Aventis Farmacêutica Ltda, Brazil) and sodium stibogluconate (Pentostan™, Glaxo Operations, UK). The second-choice drugs are amphotericin B (AmB), pentamidine, miltefosine and paromomycin, and the azole compounds including ketoconazole, fluconazole and itraconazole (WHO 2010). The antileishmanial drugs recommended by health authorities have produced severe side effects and toxicity, leading to increased use of natural products, especially those derived from plants, for leishmaniasis therapy. The problems caused by the first and second-choice drugs have generated great interest in finding alternative therapeutics with better efficacy and lower toxicity for the treatment of leishmaniasis (Rupashree & Chatterjee 2011). Essential oils (EO) have been used in traditional folk medicine to treat various diseases. The components of these oils have many different properties, including as antibacterial agents, fungicides, antiinflammatory, spasmolytic, sedative, analgesic, local anaesthetics, and in food preservation (Bakkali et al. 2008). Several reports have indicated that volatile oils from plants show potential leishmanicidal activity (Rosa et al. 2003, Ueda-Nakamura et al. 2006, Monzote et al. 2007, Santos et al. 2008a, Oliveira et al. 2009, Machado et al. 2012).

*Tetradenia riparia* (Hochstetter) Codd, a member of the family Lamiaceae, is also known as *Iboza riparia* N. E. BR., *Moschosma riparium*, or *T. riparia* (Hochstetter) N. E. BR. This shrub is widely known in South Africa and is traditionally used to treat various symptoms including fever, cough, sore throat, headache, diarrhoea, and toothache (Campbell et al. 1997). In Brazil, it is known as incense, *lavândula*, or *falsa mirra*, and is used as an ornamental (Martins et al. 2008). Isolated substances, EO, and extracts from *T. riparia* have several biological activities, including larvicidal (Weaver et al. 1992), insecticidal (Weaver et al. 1994), antimicrobial (Van Puyvelde et al. 1986), trypanocidal, antimalarial, antispasmodic (Campbell et al. 1997), antiinflammatory, and anticancer (Torquilho 2001), as a repellent against*Anopheles gambiae* (Omolo et al. 2004), and as an acaricide against *Rhipicephalus (Boophilus) microplus* (Gazim et al. 2011).

Considering the paucity of data on the effect from *T. riparia* (here termed TrEO) on *Leishmania* species and its proven efficacy against important pathogenic microorganisms, the effects of the TrEO on leishmaniasis should be explored for possible use as an alternative treatment. Also, new compounds with better efficacy and fewer adverse effects might be obtained from the TrEO. This study evaluated the activity of the TrEO obtained in different seasons against *L. (L.) amazonensis* in vitro, and in BALB/c mice to determine its potential for leishmaniasis treatment. Also, the cytotoxicity to J774.A1 and murine macrophages, and to human erythrocytes was tested in vitro. The effects of the TrEO on nitric oxide (NO) produced by murine peritoneal macrophages were also determined*.*

## MATERIALS AND METHODS

*Plant materials* - Samples of *T. riparia* leaves were collected monthly between September 2006-August 2007, and another sample in January 2012 in Umuarama, state of Paraná (PR), Brazil (23º46’22”S 53º16’73”W, 391 m elevation). The plant was identified by Prof Ezilda Jacomasi, Department of Pharmacy of Paranaense University (UNIPAR), PR. A voucher specimen is deposited in the UNIPAR Herbarium (code 2502). The TrEO was obtained from fresh leaves collected from 06:30 am-08:00 am. The samples were labelled according to the season: spring (September 23-December 21), summer (December 21-March 21), autumn (March 21-June 21) and winter (June 21-September 23).

*Extraction of the TrEO and chemical analysis* - the TrEO was extracted as described by Gazim et al. (2010). Briefly, EO was obtained from 60 g of fresh leaves by steam distillation in a Clevenger apparatus for 3 h with 600 mL of distilled water. Next, the sample of EO was collected and dried with anhydrous sodium sulphate (Na_2_SO_4_), weighed, placed in amber-coloured glass bottles and stored at 4ºC until use (Omolo et al. 2004).

*Chemical analysis of the TrEO* - The gas chromatography-mass spectrometry (GC-MS) analyses were performed using an Agilent 5973N GC-MS System operating in electron ionisation (EI) mode, equipped with a DB-5 capillary column (30 m × 0.25 mm × 0.25 μm) (Agilent, USA) was used to inject 1 μL of a solution of a sample. The initial temperature of the column was 80ºC, which was gradually raised to 260ºC at a rate of 4ºC/min. The injector (splitless, 0.5 min), and transfer line temperature were held at 260ºC and 280ºC, respectively. He (1.0 mL/min) was used as the carrier gas. Together with the sample, n-nonadecane was added as an internal standard. The same temperature program was used for GC-flame ionisation detector. The identification of the TrEO compounds was based on comparison of their retention indices (Sandra & Bicch 1987) obtained using various n-alkanes (C7-C25). Also, their EI-mass spectra were compared with the Wiley Library spectra and the literature (Adams 2007).

*Culture and maintenance of L. (L.) amazonensis* - *L. (L.) amazonensis* promastigotes (MHOM/BR/1977/LTB0016) were maintained at 25ºC; weekly subcultures were made in 199 medium (Gibco, USA), pH 7.2, supplemented with 10% foetal bovine serum (FBS) (Gibco), 1% human urine, 20 mM L-glutamine (Sigma-Aldrich Chemie, Germany), 0.063 mg/mL penicillin (Sigma-Aldrich Chemie) and 0.1 mg/mL streptomycin sulphate (Sigma Chemical Co, USA). For the antileishmanial activity, promastigotes were maintained in Schneider medium pH 7.2, supplemented with 10% FBS (Gibco) and 20 mM L-glutamine (Sigma-Aldrich Chemie) at 25ºC. For all experiments, the plates, reagents, diluents, culture medium, and plant materials were endotoxin-free.

*Antileishmanial activity* - Briefly, samples of the TrEO obtained in spring, summer, autumn and winter were solubilised in dimethyl sulfoxide (DMSO) (1.6% v/v) for the first well and serially diluted in 96-well plates (TPP^®^ test plate; Techno Plastic Products AG, Switzerland) in Schneider medium (Sigma, USA), pH 7.2 supplemented with 10% FBS and 20 mM L-glutamine. The dilutions ranged from 4.69 x 10^-3^ mg/mL to 2.40 mg/mL. A 100 mL volume of the parasite suspension (4 x 10^6^ promastigotes/mL) was added to each well of the plate. Some wells did not receive the EOs and were used as controls. The plates were incubated at 25ºC for 24 h. AmB (0.05-0.80 mg/mL) (Anforicin B; Cristália, Brazil) was used as a reference drug and internal positive control for all antileishmanial assays. Then, the parasites were counted in a Neubauer chamber and the percentage growth inhibition was calculated. The 50% inhibitory growth concentration (IC_50_)/24 h were determined by linear regression of the percentage inhibition, using a limit of statistical error of 5%. The tests were performed in triplicate (Demarchi et al. 2012).

*Haemolytic activity (HA) assay* - The potential haemolytic effects of the TrEO obtained in different seasons (spring, summer, autumn and winter) were evaluated according to Valdez et al. (2009)with some modifications. Briefly, a 3% suspension of fresh deﬁbrinated human blood (O^+^ obtained from the author) was prepared in sterile 5% glucose solution. One of several concentrations (37.5, 75, 150, 300, 600, 1,200, or 2,400 ng/mL) of each TrEO was added to each test tube and gently mixed, and the tubes incubated at 37ºC. After 1 h of incubation, the visual reading was made, and after 2 h the samples were centrifuged at 1,100 rpm for 3 min. The absorbance of the supernatant was determined at 550 nm for estimation of haemolysis. The results were expressed as percentage of haemolysis by the equation: haemolysis (%) = 100 - [(Ap-As)/(Ap-Ac) × 100], where Ap, As, and Ac are the absorbance of the positive control, test sample and negative control, respectively. AmB was used as the reference drug, Triton X-100 (Sigma-Aldrich) was used as the positive control, and the cell suspension alone was used as the negative control. The selectivity index (SI) was calculated as the proportion HA_50_/IC_50_.

*Cytotoxicity to J774.A1 macrophages* - J774.A1 macrophages were distributed in a 96-well plate (5 x 10^5^/mL/well) in RPMI-1640 medium (Gibco) supplemented with 20% FBS, 100 UI/mL penicillin G, and 0.1 mg/mL streptomycin, and incubated for 48 h at 37ºC with 5% CO_2_ for monolayer development. After the incubation, the cell monolayer was treated with different concentrations of the TrEO (4,800, 480, 300, 30, 3, or 0.3 ng/mL) from each season, and then incubated for 24 h under the conditions previously described. According toWilliams et al. (2003), cell viability was assessed using the 2,3-Bis[2-methoxy-4-nitro-5-sulfopheny]-2H-tetrazolium-5-carboxinilide (XTT) (Sigma Chemical Co) colorimetric method. A solution of XTT (100 μg/mL) and phenazine methosulfate (Sigma Chemical Co) (10 μg/mL) was added (100 µL/well) over the cell monolayer, incubated for 3-5 h at 37ºC, 5% CO_2_, and read at 450/620 nm (ASYS Expert Plus Microplate Reader; Analytical, Biochrom Ltd, UK). The experiments were performed in triplicate on different days. The data were used to obtain the 50% cytotoxic concentration (CC_50_), calculated by linear regression from the percentage of toxicity, using a statistical error limit of 5%. The SI, which indicates the degree of activity against the protozoan compared to the host cell, was calculated as CC_50_/IC_50_.

*Murine macrophage cytotoxicity* - Peritoneal macrophages were obtained from BALB/c mice (40-60 days old) euthanized by 40% CO_2_inhalation in a chamber at a moderate fill rate. This study was approved by the Ethical Committee on the Use of Experimental Animals of the State University of Maringá (UEM) (protocol 041/2011). The peritoneal cavity was washed with 5-6 mL sterile phosphate-buffered saline (PBS). Macrophage suspension was adjusted to 1 x 10^6^ macrophages/mL, and 500 µL was distributed on sterile 13 mm-diameter glass coverslips (Glasstecnica, Brazil) in 24-well culture plates (TPP test plate; Techno Plastic Products AG). After 1 h at 25ºC, nonadherent cells were removed by three washes with sterile PBS. The cultures were treated with the TrEO at concentrations from 0.002-0.2 µg/mL and incubated at 37ºC in a humid atmosphere containing 5% CO_2_ for 24 h. The coverslips were stained with 1% Trypan Blue (Sigma-Aldrich) and analysed using optical microscopy. All tests were done in duplicate, and the cytotoxicity index was expressed as percent viability. The CC_50_ (µg/mL) was defined as the dose of TrEO that reduced the viability of the macrophages by 50% compared with untreated macrophages (viability control).

*Activity against intracellular amastigotes* - The TrEO activity against intracellular amastigotes was tested using macrophages of strain J774.A1 (5 x 10^5^/500 μL). The cells were cultured on sterile glass coverslips, 13 mm in diameter, distributed in 24-well culture plates. The plates were incubated for 2 h at 37ºC in a 5% CO_2_ atmosphere. Then, promastigotes of *L. (L.) amazonensis* were added at a rate of 10 promastigotes/macrophage. After 4 h, the cultures were washed with PBS to remove nonengulfed parasites, and the macrophages were treated with 30, 3, or 0.3 ng/mL of the TrEO obtained in each season, diluted in DMSO (Sigma-Aldrich). AmB (25, 50, or 100 ng/mL) was used as a positive control and infected macrophages without treatment as a negative control. The experiments were performed in duplicate. The coverslips were removed from the plates and stained using the Fast Panoptic LB kit (Laborclin^®^, Brazil) and attached to glass slides (24 x 76 mm) with Entellan (Merck^®^, Germany). The survival index was determined by counting the number of infected J774.A1 macrophages multiplied by the mean number of parasites per macrophage.

*NO production in stimulated peritoneal macrophages with lipopolysaccharide (LPS)* - Peritoneal macrophages from BALB/c mice were obtained three days after intraperitoneal inoculation of 1 mL of thioglycolate broth. The cells were distributed in a 96-well plate (2 x 10^5^ cells/well) and incubated for 2 h at 37ºC in 5% CO_2_ atmosphere. Nonadherent cells were removed by washing with RPMI-1640 medium and treated with the TrEO obtained in autumn (0.3, 3, 30, or 300 ng/mL). After 1 h at 37ºC in 5% CO_2_, one group of cells was stimulated with 10 µg/mL of LPS *Salmonella typhimurium*(Sigma-Aldrich, Brazil). Untreated macrophages were used as a negative control. After 24 and 48 h, the supernatant was removed for the determination of nitrite levels derived from NO by the Griess method (Ding et al. 1988). The reading was performed at 450 nm (ASYS Expert Plus Microplate Reader Analytical; Biochrom Ltd). The experiments were performed in duplicate and on different days, and the results are expressed as the NO concentration (µM).

*Animals* - Female BALB/c mice approximately eight weeks of age, 20-25 g, were obtained from the Animal Facility of the UEM. Animals were maintained under standard laboratory conditions in a 12/12 h light/dark cycle with food and water*ad libitum*. The experimental protocol was approved by the Ethical Committee on the Use of Experimental Animals of the UEM (protocol 041/2011).

*Infection of animals* - BALB/c mice were anesthetised and infected subcutaneously in the right footpad with 1 x 10^6^ promastigotes of*L. (L.) amazonensis* in 40 mL of PBS, and with the same volume of PBS in the left footpad. The treatments started 30 days post-infection and continued for five weeks. The animals were monitored weekly by means of photographic records, weighing, and measurements of the thickness of the infected and uninfected footpads, using a dial gauge (Mitutoyo Corporation, Japan). The result was expressed as the difference in thickness between the parasite-inoculated footpad and the noninoculated footpad.

*TrEO treatment* - The mice were divided into groups of seven-eight animals. One group received neither infection nor treatment, and another was treated topically with 0.5% base (10% Lanette wax, 10% mineral oil, 10% propylene glycol, and purified water) containing the TrEO obtained in summer. The other groups were inoculated with *L. (L.) amazonensis* and received the following treatments: (i) topical, with 0.5% TrEO extracted in summer in the base (topical 0.5%); (ii) topical, with 1% TrEO extracted in summer with the same base (topical 1%); (iii) intraperitoneal AmB (5 mg/kg/day) in a glucose-physiological serum during 15 days (treatment control); (iv) without any treatment (positive control). Treatments started in the fourth week after infection and continued for five weeks. Fifteen days after the end of treatment, the animals were euthanized in a CO_2_ chamber.

*Parasite load in lymph nodes and spleen* - The numbers of parasites in the popliteal lymph node and spleen of the infected mice were calculated according to the method described previously by Lonardoni et al. (2000). Briefly, the popliteal lymph node and spleen were removed aseptically, weighed, and macerated in medium 199 (Gibco), pH 7.2, supplemented with 10% (v/v) FBS (Gibco), 1% human urine, 20 mM L-glutamine (Sigma-Aldrich Chemie), 100 IU/mL penicillin G (Sigma-Aldrich Chemie) and 100 μg/mL streptomycin sulphate (Sigma Chemical Co). Serial four-fold dilutions were prepared from the suspension and distributed in duplicate in a 96-well microtitre plate. After seven, 14, and 21 days of incubation at 26ºC, readings were performed and the samples were examined in an inverted microscope (Nikon, Inc) at 3,100 or 3,200 x magniﬁcation for the presence of the promastigotes. The titre was the last dilution for which the well contained at least one parasite. The parasite load (number of parasites/gram of tissue) was calculated as follows: the geometric mean of the reciprocal of the positive titres from each duplicate was divided by the weight of the lymph node or spleen. The value obtained was multiplied by the reciprocal fraction of the homogenised organ inoculated into the ﬁrst well of the culture dish. The results were compared between treated and nontreated animals.

*Statistical analysis* - The results were first analysed by the Shapiro-Wilk, Kolmogorov-Smirnov and Lilliefors test for normality. Results with a normal distribution were analysed by Student’s *t* test, and the others were analysed by the Mann-Whitney *U* test. The results were analysed by means of the software Statistica 7.0, and differences were considered significant when p < 0.05.

## RESULTS

*Chemical composition of the TrEOaccording to seasonal variation* - The TrEO obtained from all seasons contained monoterpenes, sesquiterpenes and diterpenes (hydrocarbons and oxygenated). The TrEO was extracted from 60 g of*T. riparia* leaves, with yields of 0.17-0.27%. Efficiency was highest in winter (0.27 ± 0.03%) and lowest in spring (0.17 ± 0.02%), in summer (0.22 ± 0.01%) and in autumn (0.24 ± 0.01%). The chemical composition did not differ among the seasons, but the concentration of the compounds did differ (Table I). Forty compounds were obtained from the TrEO collected during all seasons, of which 39 were identified (Table I). The oxygenated sesquiterpenes were the majority class in all seasons, with the highest concentration in winter (55.28%). The overall majority chemical compounds identified were α-cadinol and 14-hydroxy-9-epi-caryophyllene. In summer, the majority compounds were hydrocarbons and oxygenated sesquiterpenes (21.37 and 48.15%, respectively); in winter, autumn and spring were oxygenated sesquiterpenes (55.28, 45.61, and 49.44%) and oxygenated diterpenes (31.47, 25 and 23.04%).

**TABLE I:** Chemical composition of essential oil from *Tetradenia riparia*leaves of according to seasonal variation

				Composition				
				(%)				
		IRR^*b*^	IRR^*c*^					Identification
Peak	Compound^*a*^	calculate	literature	Spring	Summer	Autumn	Winter	methods
	Monoterpene hydrocarbons							
1	Limonene	1,047	1,031	0.54	3.01	T	T	*a,b,c,d*
	Oxygenated monoterpenes							
2	Fenchone	1,051	1,087	3.49	5.54	4.78	1.03	*a,b,c,d*
3	Endo-fenchol	1,093	1,112	0.59	1.10	0.72	T	*a,b,c,d*
4	Camphor	1,108	1,143	0.84	1.45	1.49	0.78	*a,b,c,d*
5	Borneol	1,119	1,165	0.52	0.21	0.59	T	*a,b,c,d*
6	α- terpineol	1,131	1,189	1.14	0.63	1.09	3.09	*a,b,c,d*
7	γ-terpeneol	1,198	1,199	0.57	0.52	T	T	*-*
	Sesquiterpene hydrocarbons							
8	δ –elemene	1,360	1,339	T	0.41	0.38	T	*a,b,c,d*
9	α-cubebene	1,336	1,345	T	0.44	T	T	*-*
10	α -Copaene	1,377	1,374	0.80	0.36	0.81	T	*a,b,c,d*
11	ß-Elemene	1,395	1,389	0.95	0.43	0.55	2.69	*a,b,c,d*
12	α -Gurjunene	1,400	1,401	0.74	0.80	0.36	T	*a,b,c,d*
13	ß-Caryophyllene	1,425	1,427	3.69	3.14	1.87	3.05	*a,b,c,d*
14	α-trans-Bergamotene	1,436	1,440	1.06	1.18	1.27	1.43	*a,b,c,d*
15	α-humulene	1,453	1,452	T	0.57	T	T	*a,b,c,d*
16	Allo-Aromadendrene	1,456	1,461	2.47	2.50	3.01	T	*a,b,c,d*
17	Germacrene-D	1,481	1,484	0.50	1.08	T	T	*a,b,c,d*
18	*Cis*-β-guaiene	1,486	1,492	0.13	0.50	T	T	*a,b,c,d*
19	Bicyclogermacrene	1,495	1,494	0.60	0.92	0.44	0.58	*a,b,c,d*
20	α-muurolene	1,502	1,500	3.45	3.31	3.65	T	*a,b,c,d*
21	*α* **-** (E,E)-farnesene	1,504	1,508	4.94	2.50	4.05	T	*a,b,c,d*
22	δ-amorphene	1,517	1,511	T	2.67	3.72	T	*a,b,c,d*
23	δ Cadinene	1,528	1,524	0.50	0.56	0.53	T	*a,b,c,d*
	Oxygenated sesquiterpenes							
24	*cis*-Muurolol-5-en-4-ß-ol	1,535	1,545	5.87	0.46	0.45	3.35	*a,b,c,d*
25	Spathulenol	1,576	1,576	1.01	0.41	T	T	*a,b,c,d*
26	Globulol	1,589	1,583	3.54	3.06	3.70	3.09	*a,b,c,d*
27	Viridiflorol	1,592	1,590	0.47	0.74	1.01	1.72	*a,b,c,d*
28	Guaiol	1,599	1,595	0.53	3.63	5.45	1.78	*a,b,c,d*
29	*epi* ***-*** *α* **-** Muurolol	1,656	1,640	3.11	T	T	T	*a,b,c,d*
30	α -Cadinol	1,669	1,645	13.81	16.91	17.16	14.82	*a,b,c,d*
31	14-hidroxi-9-epi-Caryophyllene	1,688	1,664	12.70	15.28	13.10	10.23	*a,b,c,d*
32	(2Z,6E) Farnesol	1,709	1,713	1.74	0.63	0.35	1.20	*a,b,c,d*
33	Guaiol acetate	1,716	1,724	0.89	0.72	T	2.98	*a,b,c,d*
34	14-hidroxy **-** *α* ***-*** Muurolene	1,782	1,775	1.41	T	T	8.78	*a,b,c,d*
35	8-Cedren-13-ol acetate	1,799	1,795	T	T	T	0.58	*a,b,c,d*
36	N-nonane	1,900	1,900	4.36	4.30	4.39	6.75	*a,b,c,d*
	Oxygenated diterpenes							
37	9β,13β-epoxy-7-abietene	1,988	-	7.20	5.99	7.23	9.07	*e*
	Abietatriene	2,017	2,055	0.79	T	T	T	*a,b,c,d*
	abieta-7,13-dien-18-ol	2,310	2,324	0.47	T	T	T	*a,b,c,d*
	Abietol	2,374	2,401	1.17	T	0.64	1.12	*a,b,c,d*
38	Manoyl oxide	2,421	-	0.53	T	0.63	0.81	*a,b,d*
39	Not identified	2,430	-	0.37	T	T	T	*a,b,d*
40	6,7-dehydroroyleanone	2,435	-	12.51	14.00	16.50	20.47	*e*
	Total identified	-	-	99.63	99.96	99.92	99.40	*-*
	Grouped components							
	Monoterpene hydrocarbons	-	-	0.54	3.01	-	-	*-*
	Oxygenated monoterpenes	-	-	7.15	10.45	8.67	4.90	*-*
	Sesquiterpene hydrocarbons	-	-	19.83	21.37	17.63	7.75	*-*
	Oxygenated sesquiterpenes	-	-	49.44	48.15	45.61	55.28	*-*
	Oxygenated diterpenes	-	-	23.04	19.99	25.00	31.47	*-*

*a*: compound listed in order of elution from a DB-5 column; *b*: identification based on retention index (RI); *c*: identification based on RI literature (Adams 2007); *d*: identification based on comparison of mass spectra; *e*: identification based on nuclear magnetic resonance spectra (Gazim et al. 2014); T: trace.

*Activity of the TrEO on L. (L.) amazonensis promastigotes* - The results showed that the TrEO had an inhibitory effect on the growth of *L. (L.) amazonensis* promastigotes after 24 h of treatment. The IC_50_ were 15.47 ± 4.6 ng/mL, 15.67 ± 1.70 ng/mL, 15.66 ± 2.22, and 13.31 ± 0.85 ng/mL for the oil samples obtained in the spring, summer, autumn, and winter, respectively (Table II). The IC_50_ of AmB was 41 ± 2.64 ng/mL. The TrEO samples showed similar values of IC_50_ and did not show statistically significant differences, independently of the season. The DMSO concentration used had no effect on the parasites.

**TABLE II:** Activity promastigotes forms of *Leishmania (Leishmania) amazonensis*, cytotoxicity, haemolytic activity (HA) in human blood cells and selectivity index (SI) of essential oils extracted*Tetradenia riparia* (TrEO) in different climatic periods

Drugs	IC_50_ (ng/mL) ± SE	CC_50_ (ng/mL) ± SE J774.A1	CC_50_ (ng/mL) ± SE Murine MØ	HA_50_ (ng)	SI_M1_	SI_M2_	SI_H_
TrEO							
Spring	15.47 ± 4.64	1,044.44 ± 55.55	90.94 ± 22.54	> 2,400	67.51	5.87	> 155.14
Summer	15.67 ± 1.70	1,476.00 ± 24.00	84.37 ± 5.30	> 2,400	94.19	6.01	> 153.16
Autumn	15.66 ± 2.22	391.66 ± 17.34	65.15 ± 23.20	> 2,400	25.01	1.59	> 153.25
Winter	13.31 ± 0.85	1,022.21 ± 72.85	71.25 ± 31.82	> 2,400	76.80	5.77	> 180.31
AmB	41.00 ± 2.65	ND	ND	> 2,400	ND	-	> 58.54

AmB: amphotericin B; CC_50_: 50% cytotoxic concentration of macrophages J774.A1, and evaluated by 2,3-Bis[2-methoxy-4-nitro-5-sulfopheny]-2H-tetrazolium-5-carboxinilide method; HA_50_: 50% HA; IC_50_: inhibitory concentration 50% growth; MØ: macrophage; ND: not determined; SE: standard error; SI_H_: haemolytic SI (HA_50_/IC_50_); SI_M1_: J774.A1 macrophage SI (CC_50_/IC_50_); SI_M2_: murine macrophage SI (CC_50_/IC_50_).

*Cytotoxicity of T. riparia EO* - The values for the CC_50_for J774.A1 macrophages are given in Table II. The EO obtained in summer had the highest CC_50_ (1476 ± 24.0 ng/mL) and the oil obtained in autumn had the lowest (391.66 ± 17.34 ng/mL). The SI obtained for the oil samples ranged from 25.01 (autumn) to 94.19 (summer). For murine macrophages, the TrEO showed a higher cytotoxicity compared to J774.A1 cells. For murine cells, the TrEO from spring samples showed the highest CC_50_(90.94 ± 22.54 ng/mL) and the samples from autumn the lowest (65.15 ± 23.20 ng/mL). The lowest SI was observed in autumn (1.59), and the highest in summer (6.01).

*HA assay* - The TrEO at the highest concentration tested (2.4 µg/mL) caused 2.05%, 0.63%, 4.01%, and 2.58% haemolysis for spring, summer, autumn and winter samples, respectively. AmB showed a strong haemolytic effect at 500 μg, with 61.79% haemolysis. The effect of Triton X-100, used as a positive control, was considered as 100% haemolysis (Table II).

**Fig. 1:**
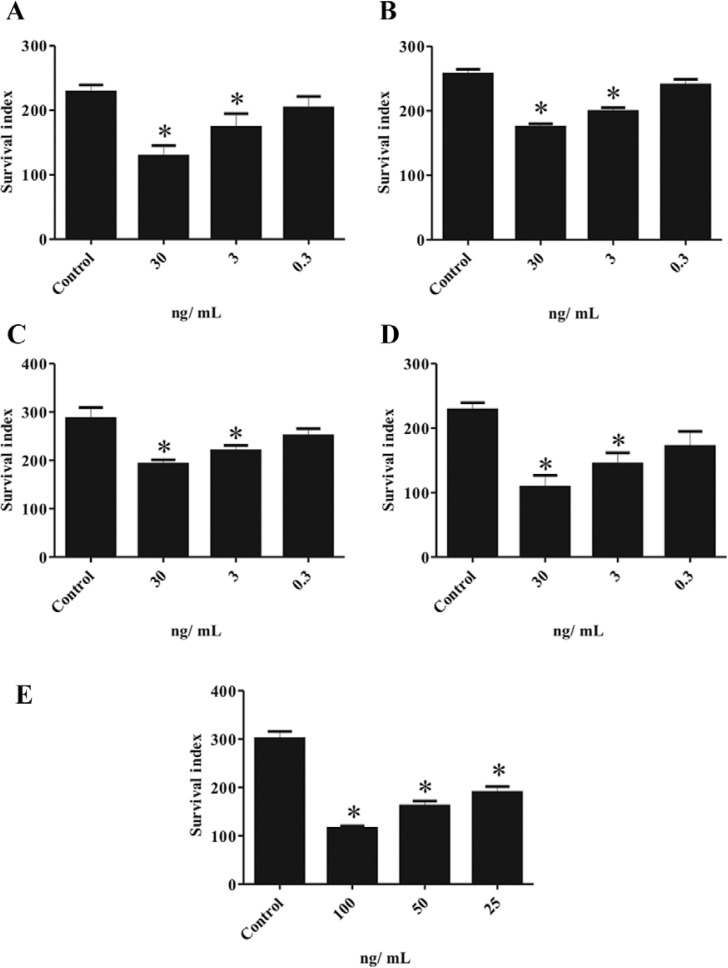
: effect of essential oil obtained from *Tetradenia riparia* (TrEO)in different seasons: spring (A), summer (B), autumn (C), winter(D), and effect of amphotericin B used as the reference drug (E). J774.A1 macrophages cultured on glass coverslips were infected with promastigotes and treated with different concentrations of the TrEO plants in different seasons. The survival index was obtained after 24 h and was determined by counting the number of infected macrophages J774.A1 multiplied by the mean number of parasites per macrophage. The data represent the mean ± standard error of the mean from three experiments performed in duplicate. Asterisk means p < 0.05.

*Activity of the TrEO on L. (L.) amazonensisintracellular amastigotes*– The TrEO obtained in different seasons significantly inhibited the survival rate of amastigotes at concentrations of 30 (p < 0.001) and 3 ng/mL (p < 0.05). These concentrations inhibited the growth of the intracellular parasites by 43.53%, 32.03%, 40.54% and 52.49%, at a concentration of 30 ng/mL. The TrEO at a concentration of 3 ng/mL caused 23.96%, 22.58%, 32.04%, and 36.81% inhibition, and at 0.3 ng/mL the percentage of inhibition was 10.96%, 7.32%, 22.45%, and 24.81% for the TrEO obtained in spring, summer, autumn and winter, respectively. AmB inhibited the survival of the *L. (L.) amazonensis* amastigotes by 61.5%, 46.28%, and 36.88% for the concentrations of 100, 50, and 25 ng/mL, respectively (Fig. 1).

**Fig. 2:**
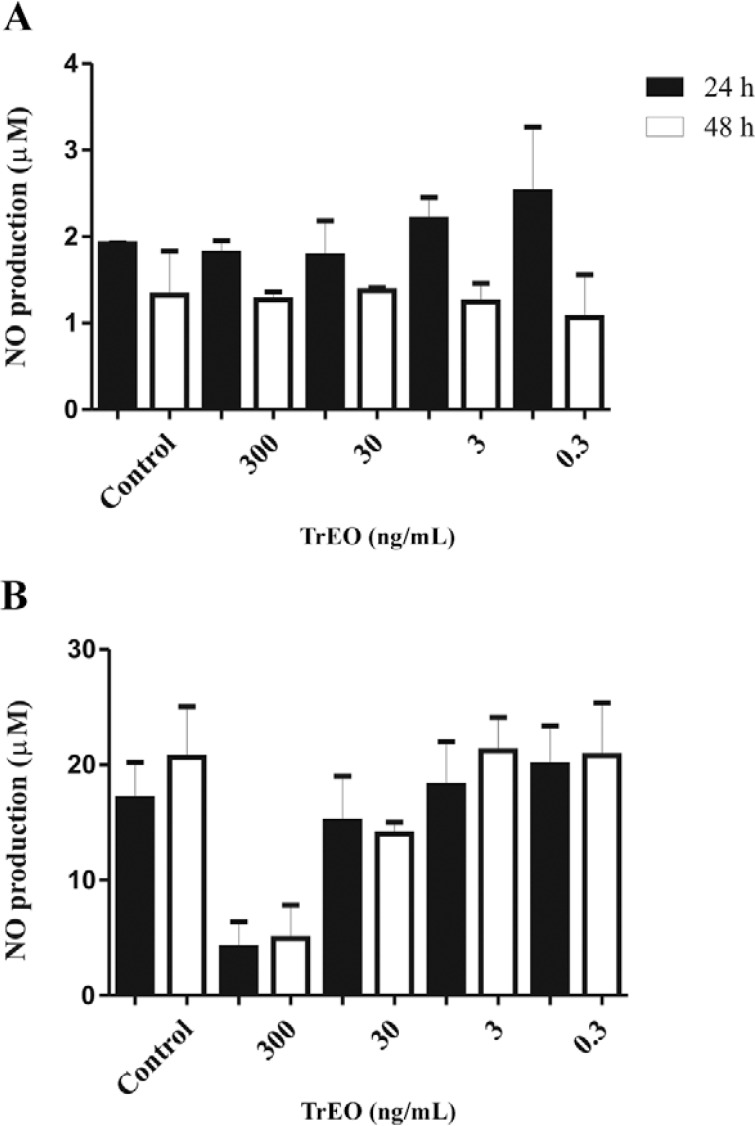
: nitric oxide (NO) production by macrophages treated with*Tetradenia riparia* essential oil (TrEO). A: NO production by macrophages treated with different concentrations of the TrEO obtained in autumn after 24 h and 48 h. The control was nontreated and nonlipopolysaccharide (LPS)-stimulated macrophages; B: NO production by macrophages stimulated with LPS (10 μg/mL) and treated with different concentrations of the essential oil after 24 h and 48 h. The control was macrophages stimulated with LPS and not treated with the TrEO. The results represent the mean ± standard error of the mean.

*NO production in stimulated peritoneal macrophages by LPS and treated with the TrEO* - Peritoneal macrophages stimulated with LPS produced significantly higher NO levels (17.07 ± 2.21 μM and 20.63 ± 3.11 μM) than nonstimulated controls (1.91 ± 0.01 and 1.32 ± 0.36 μM) after 24 and 48 h, respectively (Fig. 2). At concentrations of 300, 30, 3, and 0.3 ng/mL, macrophages stimulated with LPS produced levels of NO: 4.13 ± 1.59 μM, 15.1 ± 2.75 μM, 18.20 ± 2.69 mM, and 20.0 ± 2.40 μM after 24 h. After 48 h, the NO levels at the same concentrations were 4.93 ± 2.05 μM, 14.01 ± 0.70 μM, 21.2 ± 2.05 mM, and 20.8 ± 3.25 μM. The results demonstrated that the TrEO obtained in autumn did not significantly inhibit the production of this mediator after 24 h and 48 h, at the concentrations tested.

**Fig. 3:**
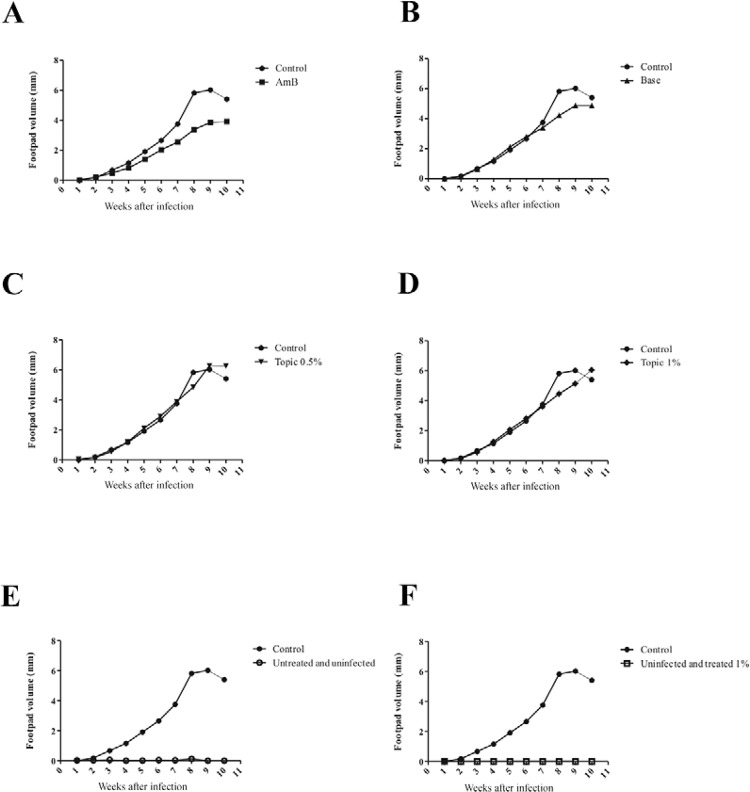
: evolution of the thickness of the paws of the animals infected with 106 promastigotes of *Leishmania (Leishmania) amazonensis* and treated with 5 mg/kg/day amphotericin B (AmB) (A), base (B), topically with 0.5% essential oil of*Tetradenia riparia* (TrEO) obtained in summer (C), and topically with 1% TrEO obtained in summer (D). The treatment was administered three times a week for 30 days after infection and continued for five weeks. E: untreated and uninfected; F: uninfected and treated.

**Fig. 4:**
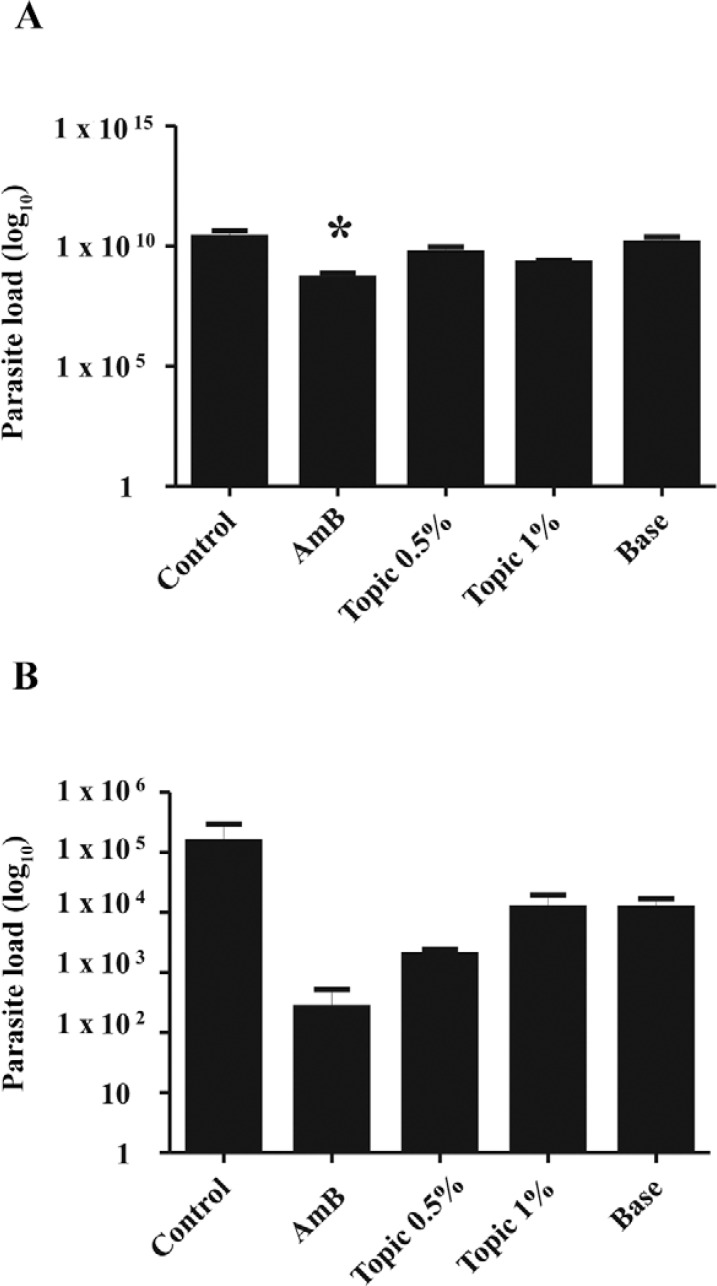
: parasite load in the popliteal lymph node (A) and spleen (B) of mice with *Leishmania (Leishmania) amazonensis* and treated with essential oil of *Tetradenia riparia*(TrEO)*.* BALB/c mice were treated with amphotericin B (AmB) (5 mg/kg/day), with base only, or topically with base plus 0.5% or 1% essential oil of the TrEO obtained in summer. Treatment started 30 days after infection and lasted five weeks. After 15 days the mice were euthanized and the parasite burdens of the lymphatic node and spleen were determined. Mann-Whitney*U* test. Asterisk means p < 0.05.

*Effects of the TrEO in vivo* - The topical treatment with the TrEO extracted in summer at concentrations of 0.5% or 1% daily for five weeks caused a reduction of the parasite load in the spleen, and the animals treated with AmB showed a significant reduction in paw thickness or parasite load after four or five weeks of treatment when compared to the control (p < 0.05) (Figs. 3, 4).

## DISCUSSION

The TREOs are a mixture of aromatic compounds, many of which have antimicrobial properties. Several studies have indicated that EOs may be an alternative for the treatment of leishmaniasis (Monzote et al. 2010). In this study, the TrEO showed inhibitory activity against promastigotes of *L. (L.) amazonensis*. In addition, this activity did not vary significantly among oil samples extracted in different seasons of the year. As suggested by the results of the present study, Gazim et al. (2010) found that the chemical constituents of the TrEO are monoterpenes, sesquiterpenes and diterpenes (hydrocarbons and oxygenates) year-round. The predominant class in all seasons was also the oxygenated sesquiterpenes, with concentrations of 64.33% in spring, 50.30% in summer, 60.21% in autumn, and 45.18% in winter.

The results showed that the chemical composition of the TrEO changed according to season and geographic location (Gazim et al. 2010). Campbell et al. (1997) also studied the chemical composition of the TrEO derived from plants in Africa, and found a different composition from the present results. Gazim et al. (2010) found monoterpenes (69%) as the main terpenoid class, and the predominant compounds were α-terpineol (22.6%), fenchone (13.6%), fenchil alcohol (10.7%), beta-caryophyllene (7.9%) and perillyl alcohol (6%). The sesquiterpenes and alcohols comprised 29.1%. Omolo et al. (2004), analysing the TrEO sampled derived from plants in Kenya, also found that monoterpenes predominated. Fenchone (64.8%) was detected among the oxygenated compounds, and limonene (2%) and 1,8-cineol (1.5%) among the hydrocarbons. In Brazil, Godoy et al. (1999) **.** evaluated the chemical composition of the TrEO derived from plants in Manaus, state of Amazonas, and also showed that monoterpenes and sesquiterpenes were the most abundant. The compounds were identified as fenchone (19.9%), 9-hydroxy-14-epi-β-caryophyllene (12.3%), α-cadinol (5.2%), camphor (3.4%) and σ-cadinene (3.1%).

The main chemical constituents of the diterpene class were predominantly 14-hydroxy-9-epi-cariophyllene (18.27-24.36%) and cis-5-muurolol en-4-beta-ol (7.06-13.78%) in winter and autumn. The majority compounds in the oxygenated diterpene class were: 9β,13β-epoxy-7-abietene (7.37%) and 6,7-dehydroroyleanone (14.89%), identified by nuclear magnetic resonance spectra and evaluated for cytotoxic, antioxidant, and analgesic activity (Gazim et al. 2014). In the present study, the TrEO from winter demonstrated a slightly higher potential leishmanial activity, showing a lower IC_50_ than those observed in the other periods of collection. Plants in winter had a higher concentration of oxygenated diterpenes such as 9β,13β-epoxy-7-abietene and 6,7-dehydroroyleanone (Table I). Demarchi et al. (2015)showed that 6,7-dehydroroyleanone from the TrEO has antileishmanial activity against promastigotes and amastigotes of *L. (L.) amazonensis*, and also found that the TrEO and its isolated compounds acted on mitochondrial and respiratory metabolism.

The sesquiterpenes are also the most common components found in copaiba oil, which also shows leishmanicidal activity against promastigotes and amastigotes of*L. (L.) amazonensis* (Santos et al. 2008a). Other studies have shown antimicrobial effects of compounds isolated from the TrEO. Van Puyvelde et al. (1986) investigated the 8(14),15-sandaracopimaradiene-7α-18-diol derived from *T. riparia*leaves, which was tested on *Staphylococcus aureus*, *Bacillus subtilis, Escherichia coli, Klebsiella pneumoniae, Pseudomonas aeruginosa, Salmonella typhymurium* and *Candida albicans.* Arruda et al. (2006) showed that limonene has activity against promastigotes of *L. (L.) amazonensis, Leishmania major, L. braziliensis* and *Leishmania chagasi*, and against amastigotes of *L. amazonensis*. This same compound also reduced the size of lesions in C57BL/6 mice infected with *L. amazonensis* (Machado et al. 2012). This study and that ofGazim et al. (2010) demonstrated that monoterpene hydrocarbons such as limonene are also present in the TrEO (3.01-3.69%), with higher concentrations in summer.

The cytotoxicity for J774.A1 of the EO samples from *T. riparia*differed according to the season when they were obtained: autumn (25.0) < spring (67.5) < winter (76.8) < summer (94.1), and for murine macrophages was: autumn (1.6) < winter (5.7) < spring (5.8) < summer (6.0). SI values less than 1 are considered to be more toxic to the host cell than to the parasite. The selectivity indices of the TrEO showed that this substance is promising for in vivo testing and has potential for treatment of this disease, especially the oil extracted in summer, which showed the highest SI. The differences in cytotoxicity and SI detected between J774.A1 and murine macrophages treated with the TrEO were also observed in other studies, but still the SI remained high. Demarchi et al. (2015) reported a similar TrEO SI (> 5) as in the present study.

Medeiros et al. (2011) found selectivity indices of 4.91 and 1.92 for thymol and for EO from *Lippia sidoides*Cham, respectively, against promastigotes forms of *L. amazonensis*. The EO from *Piper auritum* analysed byMonzote et al. (2010) showed SI of 264, 430, 166, 193 for *L. major, Leishmania mexicana, L. braziliensis*, *and Leishmania donovani*, respectively. The copaiba oil studied by Santos et al. (2008a) showed a SI of 8 for promastigotes of *L. amazonensis* in J774.A1 macrophages.

The biosynthesis of plant secondary metabolites is genetically controlled, but is also strongly influenced by environmental factors and crop storage conditions. These factors are critical in affecting the quantity and quality of the compounds (Blank et al. 2007). According to Sarma (2002), precipitation, temperature, light, and humidity all affect the overall yield and major constituents of the EO from *Cymbopogon winterianus*. In this study, the TrEO obtained from plants in autumn showed a lower SI compared to oil samples extracted in other seasons. This selectivity may result from several factors such as those mentioned above; the rainfall for the period was around 50 mm^3^ above the historical mean, according to the Agronomy Institute of Paraná (IAPAR 2012). The variation during the period when the *T. riparia*leaves were collected may indicate a possible change in the activity and cytotoxicity with the seasons.

Our results also showed that the TrEO obtained in different seasons caused haemolysis of only 2.05%, 0.63%, 4.01%, and 2.58% in spring, summer, autumn and winter, respectively, at the highest concentration tested. Several studies have demonstrated in vitro HA of herbal substances, such as EO from Karanja (Gandhi & Cherian 2000), or pharmaceuticals (Yamamoto et al. 2001). These, in turn, have been used as a method for toxicity screening assays in vitro*.*

Regarding the intracellular amastigotes, the TrEO significantly inhibited the growth of *L. (L.) amazonensis* at concentrations of 30 ng/mL (p < 0.001) and 3 ng/mL (p < 0.05). The oil extracted in winter had the highest inhibitory activity against intracellular parasites, with 52.49%, 36.81%, and 24.81% for concentrations of 30 ng/mL, 3 ng/mL and 0.3 ng/mL, respectively. The TrEO effects on*Leishmania* amastigotes may result from the predominant presence of diterpenes and calyculone (24.70%) and abietadiene (13.54%), as described byGazim et al. (2010). Other studies have shown that EOs from other plant species also have antileishmanial activity, such as*P. auritum*, which inhibited the growth of amastigotes of*L. donovani* (Monzote et al. 2010), and *L. sidoides* Cham (Santos et al. 2008b), which showed activity against *L. (L.) amazonensis.*

Recent studies of several plants EOs have shown that these components have potent biological activities, including antioxidant and antiinflammatory effects (Tsai et al. 2011). Some EOs have immunomodulatory effects, useful in the control of many infectious diseases, and have no adverse effects on the host (Antony et al. 2005).

Our results showed that the TrEO did not significantly stimulate NO production in murine resident macrophages from BALB/c mice, even in macrophages stimulated with LPS at 24 h and 48 h. The TrEO extracted in autumn was used because of its greater cytotoxicity compared to other seasons and also because the sample was larger. Demarchi et al. (2015) also found that the TrEO is not able to increase NO production, nor did 6,7-dehydroroyleanone isolated from the TrEO. NO is involved in immune processes for *Leishmania*elimination (Brunet 2001) and the absence of this mediator suggests that the EOs of this plant species do not activate these mechanisms in vitro. Still other mechanisms may be stimulated by the TrEO, but this question remains to be investigated.

For in vivo assays, we tested the TrEO obtained from summer because it was the least cytotoxic. The in vivo results showed that BALB/c mice infected with *L. (L.) amazonensis* and treated topically with the oil extracted in summer did not show a statistically significant reduction in lesion size; this may be due to the low concentration of the oil, only 0.5% and 1%. Although the lesion size was not reduced, the parasite load in the spleen decreased significantly. Thus, we suggest that other doses or concentrations, administration route, and other factors should be tested to potentially enable the use of the TrEO for leishmaniasis therapy. According to studies by Santos et al. 2008a), 4% copaiba oil tested topically and subcutaneously also did not significantly reduce lesion size. However, further studies should be conducted in our laboratory, using higher concentrations of the TrEO in in vivo experiments*.*

Our study revealed a significant activity of the TrEO against promastigotes and amastigotes of *L. (L.) amazonensis*, with no cytotoxicity to J774.A1 and murine macrophages or to human erythrocytes, regardless of the season when the oil was extracted. Based on the data obtained here, our new goals are to test a new formulation with higher concentrations of the TrEO. The present results indicate that the TrEO shows potential for development of a new and safer drug with fewer side effects for the treatment of leishmaniasis.
